# Practical Approach for Determining Material Parameters When Predicting Austenite Grain Growth under Isothermal Heat Treatment

**DOI:** 10.3390/ma16196583

**Published:** 2023-10-06

**Authors:** Mohd Kaswandee Razali, Afaf Amera Abd Ghawi, Missam Irani, Suk Hwan Chung, Jeong Muk Choi, Man Soo Joun

**Affiliations:** 1 Engineering Research Institute (ERI), School of Mechanical and Aerospace Engineering, Gyeongsang National University, Jinju 52828, Republic of Korea; mohdkaswandee@gnu.ac.kr; 2 Graduate School of Mechanical and Aerospace Engineering, Gyeongsang National University, Jinju 52828, Republic of Korea; afafamera@gnu.ac.kr; 3 Institute of Metal Forming, Technische Universität Bergakademie Freiberg, 09599 Freiberg, Germany; Missam.Irani@imf.tu-freiberg.de; 4 Metal Forming Research Corporation (MFRC), Jinju 52818, Republic of Korea; shchung@afdex.com; 5 Jinhap Co., Ltd., Daejeon 34302, Republic of Korea; superjm@jinhap.com

**Keywords:** austenite grain growth (AGG), generalized reduced gradient (GRG) optimization, isothermal heat treatment

## Abstract

An investigation of austenite grain growth (AGG) during the isothermal heat treatment of low-alloy steel is conducted. The goal is to uncover the effect of time, temperature, and initial grain size on SA508-III steel grain growth. Understanding this relationship enables the optimization of the time and temperature of the heat treatment to achieve the desired grain size in the studied steel. A modified Arrhenius model is used to model austenite grain size (AGS) growth distributions. With this model, it is possible to predict how grain size will change depending on heat treatment conditions. Then, the generalized reduced gradient (GRG) optimization method is employed under adiabatic conditions to characterize the model’s parameters, providing a more precise solution than traditional methods. With optimal model parameters, predicted AGS agree well with measured values. The model shows that AGS increases faster as temperature and time increase. Similarly, grain size grows directly in proportion to the initial grain size. The optimized parameters are then applied to a practical case study with a similar specimen size and material properties, demonstrating that our approach can efficiently and accurately predict AGS growth via GRG optimization.

## 1. Introduction

In modern nuclear power plants, ASME SA506-III steel is used in nuclear reactor pressure vessels and steam generators that serve as the critical components of nuclear power equipment and are key to the lifespan of nuclear power plants. The high strength and toughness of this steel prevent failure under severe working conditions [1,2,3]. Thus, understanding the microstructural changes that occur during the complex heating and forming processes is essential for obtaining the desired mechanical properties in heavy forgings produced from several hundred tons of steel ingots [1]. Modeling the AGS evolution during heating allows the prediction of the final mechanical properties of forged parts [4,5,6,7]. Coarse grain and uneven grain size distributions increase the brittleness of steel by effectively reducing its toughness and plasticity [7]. Therefore, studying the AGS distribution and strictly controlling AGG during heat treatment is vital.

Microstructural evolution during AGG has been simulated using various physical, empirical, and semi-theoretical models [8,9,10,11,12,13,14,15,16,17,18,19,20,21,22,23,24], as summarized in Table 1. A fundamental equation for the prediction of AGS growth during isothermal heat treatment was proposed by Beck [8,9] in the late 1940s, wherein the effect of holding time was considered in the simplest form. Hillert [10] later modified and improvise Beck’s model and revealed a limit to grain growth above a certain threshold.

Several other models are versions of Beck’s model effectively modified based on experimental data and regression analysis results. For example, in Nishizawa’s model [11], Beck’s model was further modified by considering the effects of initial grain size in the AGG of single- and dual-phase steels. Turnbull [12] and Yu [13] proposed an Arrhenius-type model that considered the effects of grain growth exponent values, activation energy, and temperature, which commonly change with heating conditions, to determine the factors driving AGG for improved AGS prediction accuracy. Sellers [14] modified the Arrhenius model by using a universal constant, replacing the initial grain size parameter, and neglecting the effect of holding time to determine AGG after the hot deformation of low-carbon steel. Lee [15] included the effects of holding time and initial grain size in a modified Arrhenius model when predicting the AGS of global low-alloy steels and investigated the effects of alloying elements on AGG; Lee’s modifications have since been applied extensively in AGG studies. For example, a similar model was developed by Xu et al. [16] for predicting the AGG of hot-rolled dual-phase steel. Duan et al. [17] used a similar approach to reveal that the AGS increases with the austenitization temperature and holding time in pipeline steel. Liu et al. [18] and Chen et al. [19] indicated that the AGG rate during austenitization decreases in different steel grades with increasing initial grain sizes. Following this, Raghunathan [20] and Anelli [21] presented the growth kinetics, according to grain size, of hot-rolled Al-Mg alloys and proved that the model can also be applied to other materials in addition to standard low-alloy steel. Subsequently, Jung et al. [22] established an average AGS method based on Lee’s modifications to study the effects of AGS distribution on the material’s mechanical properties on hot-rolled steel in a high-temperature holding process. Notably, Donati et al. [23] believed that the solution accuracy of Lee’s modifications could be further improved by adding exponential values to the initial grain size parameter; their model was implemented using DEFORM FEM code and the parameters were calculated through linear regression. Schikorra et al. [24] further verified this AGS model by analyzing the microstructural evolution of an aluminum alloy with backward extrusion with good accuracy.

Despite the advances presented in the literature review, research on the microstructural evolution and modeling of AGG in SA508-III during the heating process is scarce. Investigating AGG behavior during hot metal forming is crucial. In most cases, the traditional empirical equation, specifically the modified Arrhenius model, has been applied to mathematically describe the AGG in metallic materials [25]. Despite differences in formulation among the models, the effects of three factors, i.e., the initial grain size, holding time, and heating temperature, cannot be neglected when predicting AGS growth. Among the models presented (Table 1), that of Donati [23], which considers the effects of all critical factors on AGS evolution for steel, was utilized in the current study. In our finite element (FE) analysis, instead of universal constants, the model parameters were characterized as functions of temperature using a GRG optimization method [26,27]. The heat treatment process of undeformed axisymmetric cylindrical specimens under adiabatic conditions was investigated to reduce the complexity of the linear regression analysis, which takes considerable time to characterize. The FE prediction of the AGS over time was verified based on the test results on SA508-III steel that were found in the literature [1]. The prediction accuracy improved significantly when the optimal values were utilized in consideration of the heating temperature sensitivity [28,29,30,31]. This study simplifies, and thus enhances the practicality of, AGG research, thereby allowing AGS to be predicted with greater accuracy.

**Table 1:** Summary of the AGS growth models during the heating process.

Author	Models for AGS Growth
Beck [8,9]	$d_{G}=Kt^{m}$
Hillert [10]	$d_{G}={\exp\left(t\right)}^{m}$
Nishizawa [11]	$d_{G}={\left({d_{0}}^{m}+Kt\right)}^{1/m}$
Turnbull [12] Yu [13]	$d_{G}=d_{0}\exp\left(-\frac{Q}{RT}\right)$
Sellers [14]	$d_{G}=A\exp\left(-\frac{Q}{RT}\right)$
Lee [15] Xu [16] Duan [17] Liu [18] Chen [19] Raghunathan [20] Anelli [21] Jung [22]	$d_{G}=d_{0}+At^{m}exp\left(-\frac{Q}{RT}\right)$
Donati [23] Schikorra [24]	$d_{G}={\left[{d_{0}}^{m}+Atexp\left(-\frac{Q}{RT}\right)\right]}^{1/m}$

## 2. Materials and Experiments

**Figure 1:**
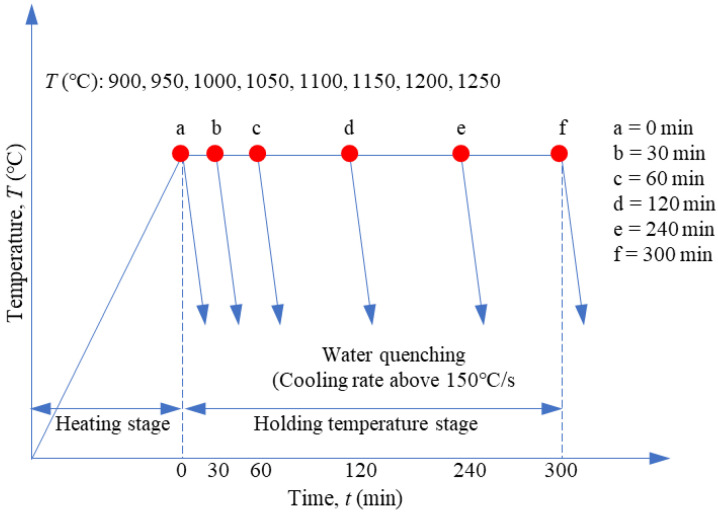
Schematic representation of the grain growth heat treatment process.

Steel alloy SA508-III with a chemical composition of 0.18% C, 0.17% Si, 1.4% Mn, 0.14% Cr, 0.51% Mo, 0.79% Ni, 0.04% Cu, 0.003% S, 0.005% P, 0.005% V, 0.022% Al, 0.013% Ti, 0.0008% Co, 0.004% As, 0.0042% Sn, and 0.0123% N (by wt.%) was investigated. The experimental data were taken from Dong et al. [1]. The cylindrical specimens (diameter: 10 mm; height: 15 mm) were heated to a specific temperature (900–1250 °C) at a heating rate of 15 °C/min and held for 0–300 min; the samples were then quenched in cold water immediately, as summarized schematically in Figure 1.

**Figure 2:**
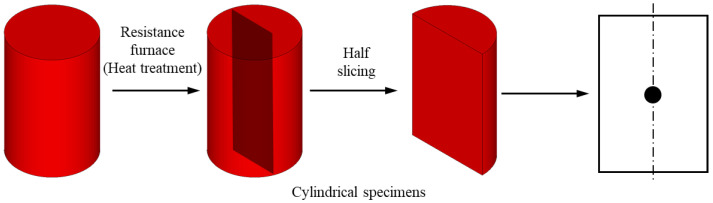
Conceptual design and the central point at the axial section for optical microstructure analysis.

**Figure 3:**
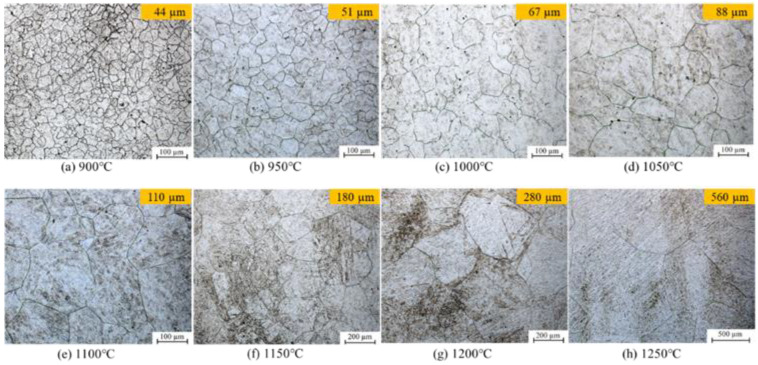
Measured morphologies of the AGS with a holding time of 30 min at different heating temperatures [1].

All specimens were then cut (axial section), polished, and etched in saturated picric acid solution. The etching time varied from 10 to 60 s to reveal visible austenite grain boundaries. AGS growth was then investigated at the central point (Figure 2) using a transmission electron microscope JEM-2100F (JEOL, Akishima, Japan). Figure 3 shows the morphologies of AGS measured at a central point after 30 min at different temperatures. The AGS increased gradually with the heating temperature. Additionally, ultrafine austenite grains were distributed homogeneously throughout the material, as shown in Figure 3a,b. However, the AGS boundaries became gradually curved and coarse as the temperature increased from 1100 °C to 1250 °C (Figure 3c–h).

Furthermore, study by Dong et al. [1] showed the AGS morphologies measured at the central point as functions of the heating temperature and holding time. In his study, the results showed exponential growth in AGS as the heating temperature increased, eventually increasing parabolically for prolonged holding times. Thus, it can be concluded that the grain growth process is normal.

## 3. Material Parameter Characterization and FE Model of AGS Growth

### 3.1. Mathematical Model of AGS Growth

As mentioned earlier, the three main factors in the AGS growth mathematical model have been identified as the initial grain size, the holding time, and the heating temperature, based on previous studies. These factors have high sensitivity and reliability, and thus cannot be ignored when making predictions. Based on the summary of previous work in Table 1, the modified model by Donati et al. [23] was selected as the AGS growth model, as given below:

(1) $$d_{G}={\left[{d_{0}}^{m}+Atexp\left(-\frac{Q}{RT}\right)\right]}^{1/m},$$

Here, $d_{0}$, *t*, and *T* are the initial grain size (μm), holding time (s), and absolute heating temperature (K), respectively. *R* is the universal gas constant (8.314 J/molK^−1^), *Q* is the apparent activation energy of grain growth (J/mol), and *A* and *m* are material constants. Note that the initial grain sizes are dependent on the heating temperature (for a holding time of 0 min). The activation energy and material constants of the selected model were characterized as functions of temperature using the GRG optimization technique, minimizing the error between the measured [1] and predicted AGS at the centroid of the specimen; here, the root-mean-square-error formula was utilized to minimize the computational time and resources required for characterizing the effects as opposed to those of complex linear regression analysis with universal constants. The objective function to be minimized was formulated as follows:

(2) $$\varphi_{o}=\sqrt{\frac{\sum_{i=1}^{N}{\left[d_{G,\mathrm{experiment}\left(i\right)}-d_{G,\mathrm{prediction}\left(i\right)}\right]}^{2}}{N}},$$

Here, *d*_G,experiment (i)_ and *d*_G,prediction (i)_ are the measured and predicted AGS values, respectively, and *N* is the number of the temperature case study at which the grain size was measured. The parameters listed in Table 2 were then acquired using a GRG optimization technique [25,26] to predict AGS growth.

**Figure 4:**
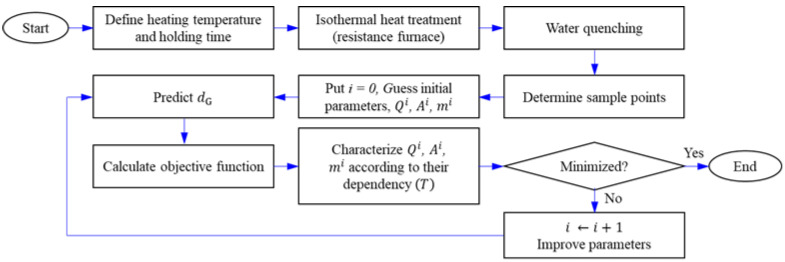
Flowchart showing how to predict *d*_G_ using the optimal material parameters.

With the proposed GRG optimization method, the material parameters can be quickly and accurately obtained without the need for complicated and time-consuming data processing, in contrast to the conventional methods used in earlier studies [15,16,17,18,19,20,21,22,23,24]. The flowchart in Figure 4 summarizes our approach.

### 3.2. Finite Element Model

**Figure 5:**
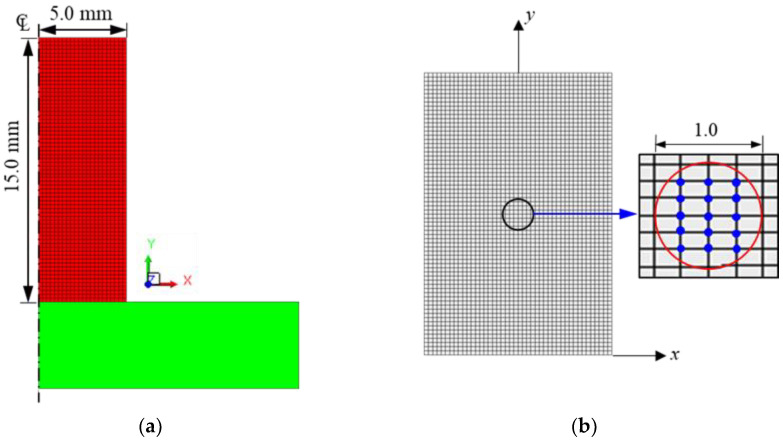
Analytical model for AGS growth predictions: (**a**) Established finite element (FE) model; (**b**) sampling location and nodal values used.

The AGS growth prediction based on Equation (1) was implemented using commercial FE software V23R02 [25]. Figure 5a illustrates an axisymmetric FE model of cylindrical specimens used in a heat treatment test, carried out under adiabatic conditions with 3000 quadrilateral elements. Remeshing was deactivated to minimize the error due to the numerical smoothing of state variables. The Coulomb friction coefficient of 0.2 was assumed at the interface between the tool and specimen [1,28,30] even though no deformation occurred. The specimen and dies were assumed to behave as thermoviscoplastic and rigid materials, respectively. A series of simulations were conducted at various heating temperatures (900, 950, 1000, 1050, 1100, 1150, and 1200 °C) and holding times (0, 30, 60, 120, 240, and 300 min). The AGS was measured at the central point on the plane section, as illustrated in Figure 5b. The central point was selected, given that the AGSs over all plane sections shared almost the same values. The AGS calculated at the central point is the mean nodal value in a small measuring circle (diameter of 1000 μm) [26,30], i.e., the mean value of the AGS prediction for 15 nodes, representing the *d*_G_ prediction at the sample point. The central points for each combination of heating temperature and holding time were used as control points for both FE predictions and optimal material identification.

## 4. Results and Verification

### 4.1. Method Validation

**Figure 6:**
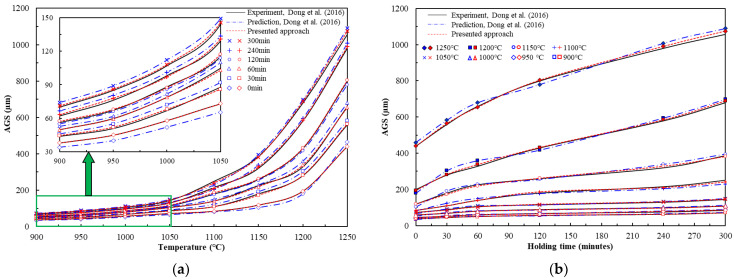
AGS after heat treatment at various heating temperatures and holding times [1]: (**a**) Heating temperatures; (**b**) holding times.

Predicted and measured AGS growth for the steel were compared across different experimental scenarios, as shown in Figure 6. Our modified characterization model showed better accuracy than that presented by Dong et al. [1], although the latter provided experimental curves with an acceptable fit. The correlation between the fitted *d*_G_ values of the presented approach and that of the measured AGS growth was higher than that found by Dong et al. [1], with a maximum error of 3.5% (standard deviation: ±2.2%) compared with 10.5% ± 2.8%, respectively. The approach presented here is simpler and more practical than that followed by Dong et al., being applicable to most cases of grain size microstructure evolution that are best characterized using a GRG optimization technique.

**Figure 7:**
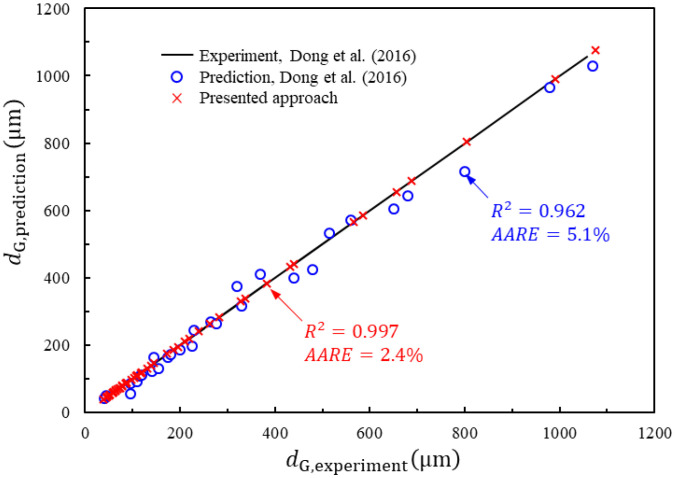
Comparisons of *d*_G_ between experiments from Dong et al. [1] and the proposed approach.

The errors of the two fitting approaches are shown in Figure 7. The predictability of the presented approach can be quantified by comparing standard statistical parameters such as the average absolute relative error (*AARE*) and coefficient of determination (*R*^2^). The *AARE* is calculated by comparing the relative errors between each term in the equation and is therefore an unbiased statistical parameter. *R*^2^ measures the linearity between the experimental and predicted values. The *AARE* and *R*^2^ are calculated as follows:

(3) $$AARE=\frac{1}{N}\sum_{i=1}^{N}\left|\frac{d_{G,\mathrm{experiment}(i)}-d_{G,\mathrm{prediction}(i)}}{d_{G,\mathrm{experiment}(i)}}\right|\times 100\%,$$

(4) $$R^{2}=1-\frac{\sum_{i=1}^{N}{\left[d_{G,\mathrm{experiment}(i)}-d_{G,\mathrm{prediction}(i)}\right]}^{2}}{\sum_{i=1}^{N}{\left[d_{G,\mathrm{experiment}(i)}-{\bar{d}}_{G,\mathrm{experiment}(i)}\right]}^{2}},$$

Here, ${\bar{d}}_{G,\mathrm{experiment}(i)}$ is the mean value of measured AGS growth. The *AARE* and *R*^2^ values of $d_{G,\mathrm{prediction}}$ when using the proposed approach (*AARE* = 2.4% and *R*^2^ = 0.997) are slightly lower than those found by Dong et al. (*AARE* = 5.1% and *R*^2^ = 0.962). Thus, although both approaches are capable of making good predictions, the superiority of our approach reflects not only high solution accuracy but also greater practicality, given that the GRG optimization technique is much simpler and more reliable. Hence, our approach for modeling AGS growth is valid.

**Figure 8:**
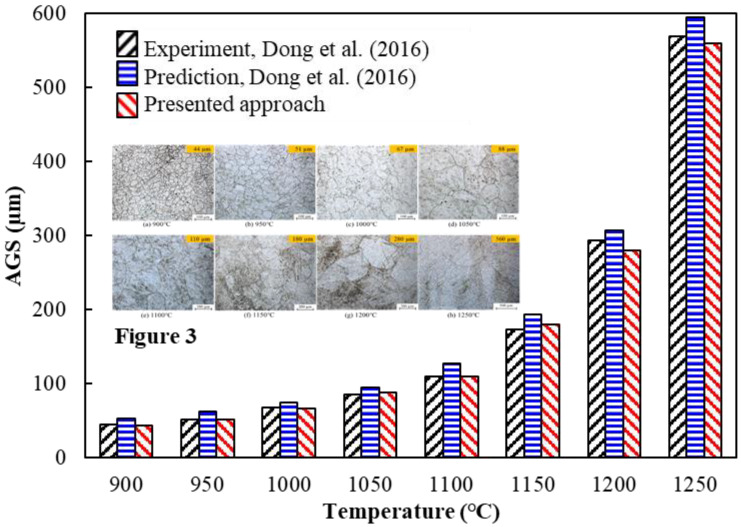
Comparison of AGS growth with a holding time of 30 min at different heating temperatures [1].

Figure 8 shows the comparison of the AGS values of the steel at different heating temperatures for a holding time of 30 min. The AGS increased steadily with the heating temperature and was equally distributed and uniform over the AGS range of 30–600 μm and the heating temperature range of 900–1250 °C. At 900 °C, 950 °C, 1000 °C, and 1050 °C, the predicted AGS values were 45, 52, 68, and 86 μm, respectively; these values were slightly higher compared with *d*_0_ (Table 2), with approximately 15% growth for each case. Despite this, the AGS began to change dramatically as the heating temperature approached 1100 °C, 1150 °C, 1200 °C, and 1250 °C, at which points the predicted AGS values were 110, 173, 282, and 565 μm, with growth rates of 30%, 44%, 46%, and 28%, respectively. The AGS predictions were similar to the measured AGS values reported in Figure 3a–h. With this, it can be concluded that the FE predictions of AGS growth in this study were almost identical to the experimental data over the entire range of interest.

### 4.2. Application Example

A Gleeble test was used by Dong et al. [1]; in this, the sample was heated to 1200 °C at a heating rate of 15 °C/min, held for 30 min at ambient temperature, and then immediately quenched in cold water. Metallographic observations were then conducted on the specimen after wire cutting, polishing, and picric acid etching.

**Figure 9:**
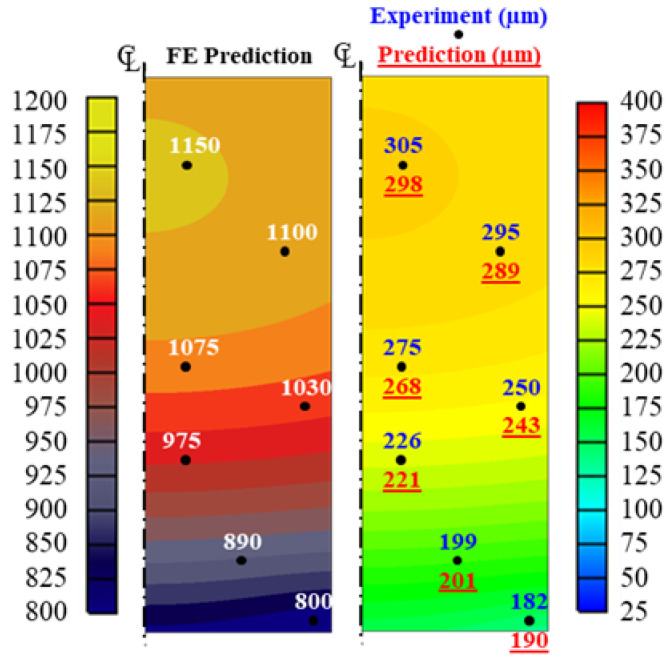
Comparison of experiment and predicted AGS after a holding time of 30 min with an initial temperature of 1200 °C.

The optimized parameter characterization method described in the previous section was then applied to a practical case study with a similar specimen size and material properties. Based on the optimized parameters, the AGS prediction and its corresponding temperature were compared with the experimental results at certain sampling points, as shown in Figure 9. The AGS at the lower surface had a low growth rate as the temperature dropped faster than the core, which had a high growth rate. Upon sampling at high temperatures, the grains of austenite became coarser. The predicted AGS was 298 μm at a sample point of 1150 °C, which was about 1.5-fold larger than that at sample point 800 °C after a 30 min holding time. This was due to lower dislocation density at high temperatures, which leads to the growth of strain-free grains while, in contrast, lower temperatures have higher dislocation density [32,33]. Therefore, this phenomenon results in the unequal growth in AGS in high- and low-temperature areas [34,35,36,37]. Based on these circumstances, temperature has a greater influence on grain growth than time. In conclusion, the predicted and measured AGS values showed excellent agreement, with an *AARE* of 2.5%, which is sufficient for practical use.

## 5. Conclusions

The present paper describes a new method for determining AGS growth parameters. The method uses an FE model, experimental results, and optimization techniques. Optimized AGS growth parameters were acquired iteratively by minimizing the objective function of errors between target and predicted grain sizes at various sampling points. In this way, the three main AGS growth parameters in the analytical model could be systematically obtained and characterized as functions of heating temperature. The method is more accurate than the conventional way of determining the parameters as more sampling points are involved in the optimization, in which the objective function is the mean error between the predicted and measured grain size at the sampling points. Thus, the simulation can accurately predict AGS growth when the GRG optimization scheme acquires the parameters.

AGS growth predictions and measured growth were then compared across different experimental scenarios. Although Dong et al. [1] provided an acceptable fit compared with the experimental curves, our modified characterization model proved to be more accurate. Based on the present approach, the fitted *d*_G_ values and the measured AGS growth are more closely correlated than those presented by Dong et al. [1], with a maximum error of 3.5% (standard deviation = ± 2.2%, *AARE* = 2.4%, *R*^2^ = 0.997) compared with 10.5% (standard deviation = ± 2.8%, *AARE* = 5.1%, *R*^2^ = 0.962), respectively. In contrast to previous works, the approach presented here is simpler and more practical and can be used to show that grain size evolution is best described using GRG optimization.

A practical case study of optimized AGS parameters was also conducted. High heating temperatures provided rapid AGS growth compared to lower temperatures. This was attributed to the growth of strain-free grains at higher temperatures, which minimized the dislocation density, showing that temperature has a greater effect on grain growth than time. Based on our results, the predicted and measured AGS values were in good agreement, with an *AARE* of 2.5%.

## Figures and Tables

**Table 2:** *d*_G_ parameters (measured and optimized) with respect to temperature.

*T* (°C)	Measured Parameter [1]	Optimized *d*_G_ Parameters		
	*d*_0_ (μm)	*Q* (J/mol)	*A*	*m*
900	38.0	89,768	17,732	2.501
950	45.0	88,810	18,732	2.505
1000	58.0	69,550	19,732	2.848
1050	73.0	44,120	21,931	3.200
1100	85.0	32,094	23,702	3.145
1150	119.0	22,093	33,197	3.149
1200	200.0	22,000	33,200	2.904
1250	440.0	12,095	34,181	2.782
900	38.0	89,768	17,732	2.501

## Data Availability

Not applicable.
